# Evolution and function analysis of auxin response factors reveal the molecular basis of the developed root system of *Zygophyllum xanthoxylum*

**DOI:** 10.1186/s12870-023-04717-7

**Published:** 2024-02-02

**Authors:** Ying Xing, Chunli Liu, Chuan Zheng, Hong Li, Hongju Yin

**Affiliations:** State Key Laboratory of Herbage Improvement and Grassland Agro-Ecosystems; Key Laboratory of Grassland Livestock Industry Innovation, Ministry of Agriculture and Rural Affairs, College of Pastoral Agriculture Science and Technology, Lanzhou University, Lanzhou, 730000 People’s Republic of China

**Keywords:** *Zygophyllum xanthoxylum*, Lateral roots, Auxin response factors, ZxARFs, A-ZxARFs

## Abstract

**Background:**

As a xerophytic shrub, forming developed root system dominated with lateral roots is one of the effective strategies for *Zygophyllum xanthoxylum* to adapt to desert habitat. However, the molecular mechanism of lateral root formation in *Z. xanthoxylum* is still unclear. Auxin response factors (ARFs) are a master family of transcription factors (TFs) in auxin-mediated biological processes including root growth and development.

**Results:**

Here, to determine the relationship between ARFs and root system formation in *Z. xanthoxylum*, a total of 30 potential *ZxARF* genes were first identified, and their classifications, evolutionary relationships, duplication events and conserved domains were characterized. 107 ARF protein sequences from alga to higher plant species including *Z. xanthoxylum* are split into A, B, and C 3 Clades, consisting with previous studies. The comparative analysis of ARFs between xerophytes and mesophytes showed that A-ARFs of xerophytes expanded considerably more than that of mesophytes. Furthermore, in this Clade, ZxARF5b and ZxARF8b have lost the important B3 DNA-binding domain partly and completely, suggesting both two proteins may be more functional in activating transcription by dimerization with AUX/IAA repressors. qRT-PCR results showed that all *A-ZxARFs* are high expressed in the roots of *Z. xanthoxylum*, and they were significantly induced by drought stress. Among these *A-ZxARFs*, the over-expression assay showed that *ZxARF7c* and *ZxARF7d* play positive roles in lateral root formation.

**Conclusion:**

This study provided the first comprehensive overview of *ZxARFs* and highlighted the importance of *A-ZxARFs* in the lateral root development.

**Supplementary Information:**

The online version contains supplementary material available at 10.1186/s12870-023-04717-7.

## Introduction

Roots are the frontline organs in contact with environmental signals in soil, and play essential roles in whole plant growth and development. The root system of higher plants consists of an embryonic primary root and postembryonic lateral roots and adventitious roots. In dicot plants, lateral roots are remarkably plastic in adapting to ever-changing growth conditions, which is vital for expanding root system to absorb water and nutrients, and even to survival [1].

It is well known that auxin plays essential roles in almost all stages of root development [2, 3]. Previous studies about the model plant *Arabidopsis thaliana* (Arabidopsis) have identified a major auxin signaling pathway consisting of transport inhibitor response 1/auxin signaling F-box protein (TIR1/AFB1-5) receptors, auxin/indole-3-aceticacid (Aux/IAA) repressors and auxin response factor (ARF) transcription factors (TFs) [4, 5]. As the central players of auxin signaling pathway, ARFs regulate plant growth and development by regulating the expression of early/primary auxin response genes [4].

Since *AtARF1* was first identified from Arabidopsis in 1997 [6], *ARF* gene families of many species have been characterized. For instance, 3 *ARFs* in *Marchantia polymorpha* [7], 23 *ARFs* in Arabidopsis [8], 19 *ARFs* in *Punica granatum* [9], 19 *ARFs* in *Citrus sinensis* [10], 39 *ARFs* in *Litchi chinensis* [11], and 40 *ARFs* in *Medicago truncatula* [12] have been identified through genome-wide data mining. Despite undergoing hundreds of millions of years of evolution, ARF families still maintain unique conserved domains across plants [13, 14]. A typical ARF protein is characterized by a highly conserved N-terminal DNA-binding domain (DBD) that includes a B3 domain and an ARF domain, a middle region (MR) with variable sequences dividing the TFs as an activator or repressor, and two conserved C-terminal domains (CTD) [4, 13]. The two CTDs form a Phox and Bem1p (PB1) domain, which often confers interaction specificity in highly redundant protein scaffolds to facilitate signaling events [4]. This domain also presents in most of the Aux/IAA proteins, and thus named AUX/IAA domain [4]. As transcription factors, ARFs are located in the nucleus and their localization signals are in DBDs.

According to the phylogenetic analysis and predicts, ARF proteins are divided into A (A-ARFs, ARF5/6/7/8/19), B (B-ARFs, ARF1/2/3/4/9) and C (C-ARFs, ARF10/16/17) Clades with diverse roles in plants [13], which have been extensively detailed by studying on Arabidopsis. A-ARFs, as a class of activated TFs, played important roles in the process of lateral root and adventitious root development in Arabidopsis [15, 16]. The single loss-of-function mutants *arf5*, *arf6* and *arf7* and the double mutants *arf6 arf8* and *arf7 arf19* all showed different degrees of defects in lateral root and adventitious root development [16–19]. Particularly, the *arf7 arf19* double knockout mutant was severely impaired in lateral root formation [17]. For B-ARFs, studies have revealed that the knockout mutant of *ARF3* produced severe defects in carpel development, and *arf3 arf4* double mutant showed a breakdown of abaxial tissue specification in all lateral organs [20]. In addition, several C-ARFs positively regulated the formation of nitrogen-fixing nodule by regulating the balance of auxin and cytokinin in legumes [21]. Arabidopsis *ARF10/16* played key roles in the differentiation of root-stem cells [22]. Moreover, there are some *ARF* genes that seem to be involved in plant responses to stresses. For instance, phosphorylation of ARF2 modulated the expression of the K^+^ transporter gene HAK5 (high affinity K^+^ transporter 5) in response to low potassium stress in Arabidopsis [23]. SUMO (small ubiquitin-like modifier)-dependent regulation of ARF7 controlled root branching pattern in response to water availability [24]. Chinese cherry (*Cerasus pseudocerasus*) *CpARF7* participated in root development and responded to drought and low phosphorus stresses [25].

As mentioned above, most of these studies have focused on mesophytes, whereas *ARFs* in desert xerophytes with special strategies and excellent tolerance to environmental stresses remain largely uncharacterized. As a succulent xerophyte, *Zygophyllum xanthoxylum* has a developed root system dominated with robust primary root as well as well-developed lateral and adventitious roots. Especially, developed lateral roots confers *Z. xanthoxylum* strong ability for absorbing water and nutrients, which is one of the effective strategies to adapt to the drought and barren desert habitat [26, 27]. However, the molecular mechanism of lateral root formation in *Z. xanthoxylum* is elusive.

Given that identification of gene families from distinct model plants is a necessary step in formulating better hypotheses related to physiological and developmental characteristics [28], in this study, *ZxARF* gene family were firstly identified on the basis of the whole genome sequences of *Z. xanthoxylum* (PRJNA933961), and comparative evolution analyses of ZxARF and ARF protein sequences of some representative species were presented to elucidate their evolution and function related to plant growth and development. In addition, the role of *ZxARFs* in lateral root development was further confirmed by over-expression assay in Arabidopsis.

## Results

### 30* ZxARF* genes were identified in *Z. xanthoxylum* genome

We firstly isolated the complete arrays of ZxARF proteins by using the 23 ARF sequences of Arabidopsis (AtARF) as queries to blast the database of the annotated *Z. xanthoxylum* genome. After redundant result elimination and further conserved domain validation, a total of 30 potential *ZxARF* genes were finally identified and were named as detailed in Table 1 according to their homologues in Arabidopsis (Table 1).

**Table 1:** The protein characteristics of ZxARFs in *Z. xanthoxylum*

Gene Name	Gene ID	Length(aa)	ORF(bp)	Extrons	Mw(KDa)	pI	localization
*ZxARF1*	*Zx11G001712*	673	2022	14	74.86	5.81	Nucleus
*ZxARF2a*	*Zx11G001739*	716	2151	12	79.86	8.31	Nucleus
*ZxARF2b*	*Zx07G000086*	844	2535	14	94.58	6.30	Nucleus
*ZxARF3a*	*Zx02G002313*	679	2040	10	74.98	6.53	Nucleus
*ZxARF3b*	*Zx06G000685*	690	2073	10	76.46	6.56	Nucleus
*ZxARF4a*	*Zx10G001074*	826	2481	11	92.43	7.24	Nucleus
*ZxARF4b*	*Zx03G000375*	821	2466	13	92.73	6.09	Nucleus
*ZxARF4c*	*Zx03G000378*	802	2409	13	90.42	5.90	Nucleus
*ZxARF5a*	*Zx05G000609*	923	2772	15	101.47	5.45	Nucleus
*ZxARF5b*	*Zx09G000738*	815	2448	11	89.71	5.59	Nucleus
*ZxARF5c*	*Zygxa0016218*	691	2976	11	76.33	5.16	Nucleus
*ZxARF6a*	*Zx04G000410*	885	2658	15	98.14	6.05	Nucleus
*ZxARF6b*	*Zx11G000222*	899	2700	13	99.95	5.87	Nucleus
*ZxARF6c*	*Zx07G001381*	905	2718	14	100.63	6.01	Nucleus
*ZxARF7a*	*Zx01G003579*	1119	3360	14	123.14	6.60	Nucleus
*ZxARF7b*	*Zx03G001013*	1080	3243	13	120.99	6.34	Nucleus
*ZxARF7c*	*Zx01G002826*	1023	3072	13	121.28	6.48	Nucleus
*ZxARF7d*	*Zx04G002360*	1081	3246	13	120.53	6.02	Nucleus
*ZxARF8a*	*Zx08G001072*	909	2730	14	100.40	5.83	Nucleus
*ZxARF8b*	*Zygxa0018977*	661	1986	8	72.41	5.54	Nucleus
*ZxARF9a*	*Zx06G001674*	669	2010	13	74.52	5.99	Nucleus
*ZxARF9b*	*Zx02G001849*	670	2013	13	74.75	5.79	Nucleus
*ZxARF10a*	*Zx10G001143*	712	2139	4	77.95	7.53	Nucleus
*ZxARF10b*	*Zx03G000471*	724	2175	4	79.60	7.80	Nucleus
*ZxARF11*	*Zx06G002012*	653	1962	14	73.42	8.24	Nucleus
*ZxARF16a*	*Zx03G001850*	684	2055	3	75.69	6.48	Nucleus
*ZxARF16b*	*Zx02G000996*	607	1824	2	67.22	7.84	Nucleus
*ZxARF17a*	*Zx08G002186*	369	1110	2	40.28	5.31	Nucleus
*ZxARF17b*	*Zx05G001843*	584	1755	2	64.47	7.99	Nucleus
*ZxARF18*	*Zx01G002456*	659	1980	14	73.06	6.67	Nucleus

Detailed information of the predicted 30 *ZxARF* genes were further analyzed. The results showed that ORF length of these *ZxARF* genes range from 1110 bp (*ZxARF17a*) to 3360 bp (*ZxARF7a*) (Table 1), and the numbers of exon of these genes range from 2 (*ZxARF16b*, *17a*, *17b*) to 15 (*ZxARF5a*, *6a*). (Table 1). Correspondingly, the length and molecular weight vary significantly among these ZxARF proteins. ZxARF7a is the largest protein containing 1119 amino acids, whereas ZxARF17a is the smallest one, which is consist of 369 amino acids (Table 1). And the molecular weights of these proteins range from 40.28 kDa (ZxARF17a) to 123.14 kDa (ZxARF7a) (Table 1). In addition, the theoretical pI values of ZxARFs range from 5.16 (ZxARF5c) to 8.31 (ZxARF2a). The pI values of 23 ZxARFs are smaller than 7, indicating they are acidic protein, and the rest of 7 ZxARFs are alkaline (pI > 7) (Table 1). All the data collected suggested a high variability among the *ZxARF* genes in the *Z. xanthoxylum* genome. Furthermore, Plant-mPLoc software was used to predict the probable protein localization of ZxARFs. As TFs, all of ZxARFs contain a nucleus-targeting signal (Table 1), suggesting they are nuclear localization proteins.

### Phylogenetic analysis of ZxARFs and AtARFs

**Fig. 1:**
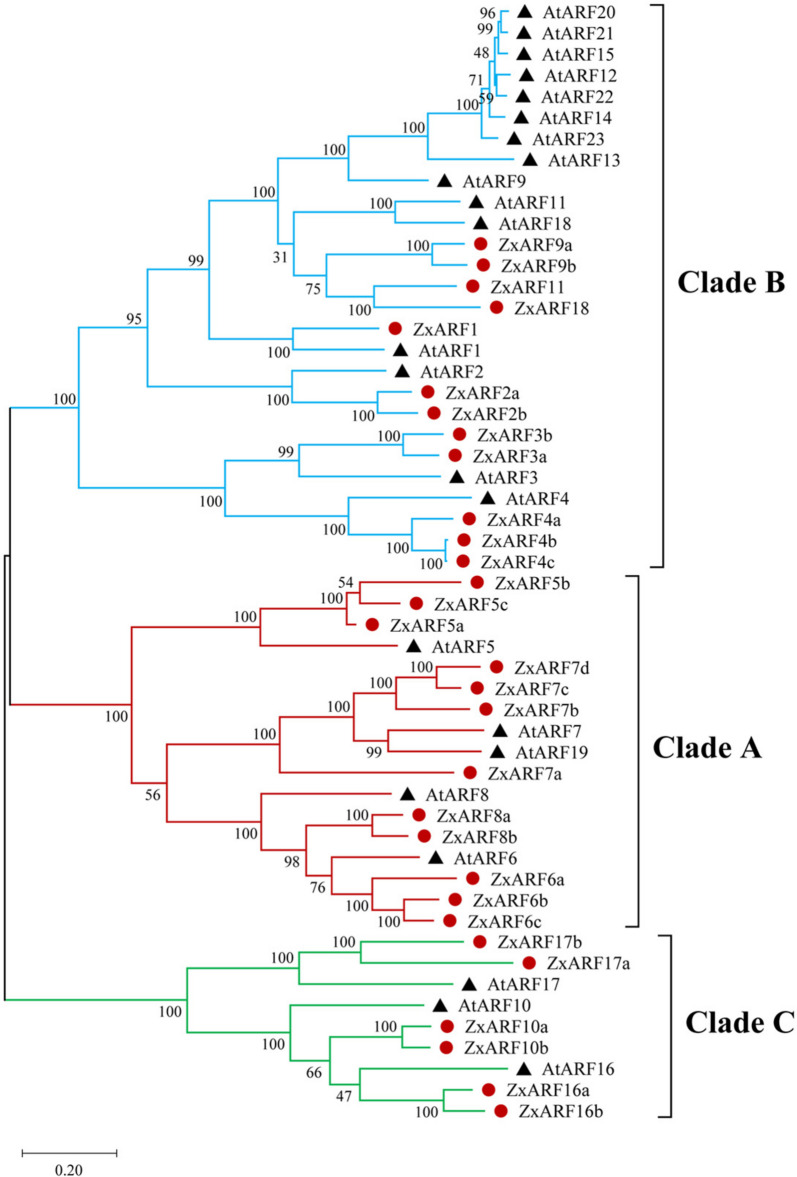
Phylogenetic tree analysis of ARFs from *Z. xanthoxylum* and Arabidopsis. In total, 30 ARFs of *Z. xanthoxylum* and 23 ARFs of Arabidopsis were used for constructing an unrooted tree via the maximum likelihood method with 1000 bootstrap replications by MEGA-X. These ARFs divided into 3 Clades (A-C), which were exhibited in red, blue, and green, respectively. The red solid circles represent ZxARFs and the black solid triangles represent AtARFs

Phylogenetic analysis plays an important role in functional predictions of various genes across species [29, 30]. To study the phylogenetic relationships between the members of *ZxARF* and *AtARF* genes, an unrooted phylogenetic tree was constructed from an alignment of the corresponding protein sequences and viewed in MEGA-X program by the maximum likelihood method (Fig. 1). It was showed that the phylogenetic tree fell into three broad groups (Clade A, B, and C) with well-supported bootstrap values. There were 12 *ZxARFs* and 5 *AtARFs* in Clade A, 12 *ZxARFs* and 15 *AtARFs* in Clade B, 6 *ZxARFs* and 3 *AtARFs* in Clade C. Interestingly, the ratio of *ZxARFs* and *AtARFs* in Clade A was 2.4:1. This result is consistent with the developed root system of *Z. xanthoxylum*, since *A-ARFs* positively regulate lateral root development [15]. Also, the number of *C-ZxARFs* is two fold of *C-AtARFs*, which may be associate with the root development of *Z. xanthoxylum* according to the previous study of Arabidopsis [22].

### Evolution analysis of ARF proteins

**Fig. 2:**
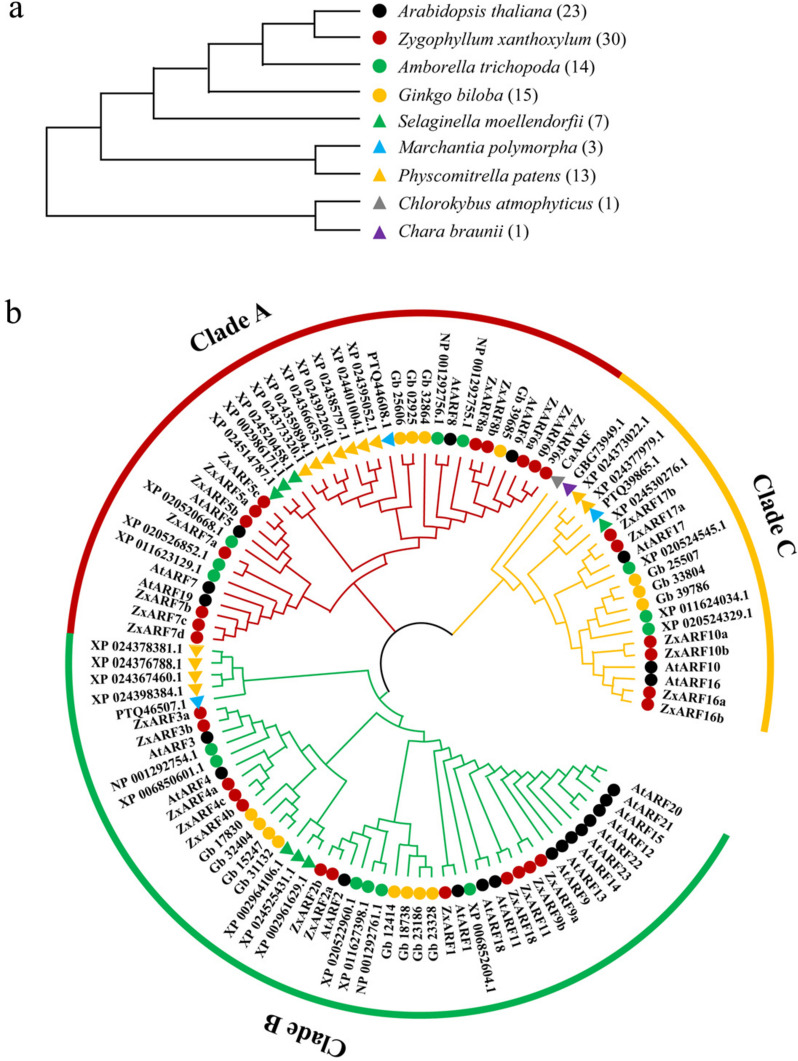
Phylogenetic trees of plants and their ARF family members. **a** Phylogenetic trees of the representative species in plant evolutionary history. The evolution analysis of *C. atmophyticus*, *C. braunii*, *M. polymorpha*, *P. patens*, *S.moellendorfii*, *G. biloba*, *A. trichopoda*, Arabidopsis, and *Z. xanthoxylum* were performed using the ANGIOSPERM PHYLOGENY WEBSITE. The solid triangles represent spore plants and the solid circles represent seed plants. **b** Phylogenetic analysis of ARF family members of plants in **a**. ARF amino acids sequences were used for constructing an unrooted tree using the maximum likelihood method with 1000 bootstrap replications by MEGA-X. In total, 107 ARFs were analyzed and divided into 3 Clades (A-C), which were exhibited in red, green, and orange, respectively. Distinctive species and the corresponding proteins are indicated with different colored symbols as Fig. 2a

The origin and adaptive evolution are vital to predict the function of genes [31]. To elucidate the phylogenetic relationships and evolutionary history of plant *ARF* genes, the phylogenetic tree was constructed by a maximum likelihood method, based on 107 ARF protein sequences from alga to higher plant species, including *Chlorokybus atmophyticus* (1), *Chara braunii* (1),* M. polymorpha* (3), *Physcomitrella patens* (13), *Selaginella moellendorfii* (7), *Ginkgo biloba* (15), *Amborella trichopoda* (14), Arabidopsis (23) and* Z. xanthoxylum* (30) (Fig. 2a, b). ARF family members in* C. braunii* and *A. trichopoda* were firstly identified in this study (Fig. 2b). Meanwhile, except that the members of ARFs in *S. moellendorfii* are only 7, which is different from the reports of Jo et al. [32], the else are in line with previous studies [7, 8, 14, 33, 34] (Fig. 2b).

Phylogenetic analysis showed that these ARF proteins were also split into three Clades: Clade A-C. ARF proteins of the 2 chlorophytes were only located in Clade C, whereas ARF proteins of bryophytes were distributed in Clade A-C. These results indicated that Clade C probably be the origin of ARF proteins in land plants, as well as that ARF proteins in Clade A and B may play vital roles in the evolution of land plants. The phylogenetic tree also showed that CaARF is the most ancient ARF protein, which is in line with the previous study [14]. ARF family members had expanded largely in the process of evolution. Among them, there were no A-ARF members in the rootless algae, while the number of A-ZxARFs was the largest among the plants (Fig. 2b and Table 2). It further demonstrates that the expansion of A-ZxARFs may be an important reason for the formation of the developed root system in *Z. xanthoxylum*.

**Table 2:** The number of plant ARF family proteins

Species	Clade A	Clade B	Clade C
*C. atmophyticus*	0	0	1
*C. braunii*	0	0	1
*M. polymorpha*	1	1	1
*P. patens*	7	4	2
*S. moellendorfii*	3	3	1
*G. biloba*	4	8	3
*A. trichopoda*	5	6	3
Arabidopsis	5	15	3
*Z. xanthoxylum*	12	12	6

### Comparative analysis of ARF proteins between xerophytes and mesophytes

**Fig. 3:**
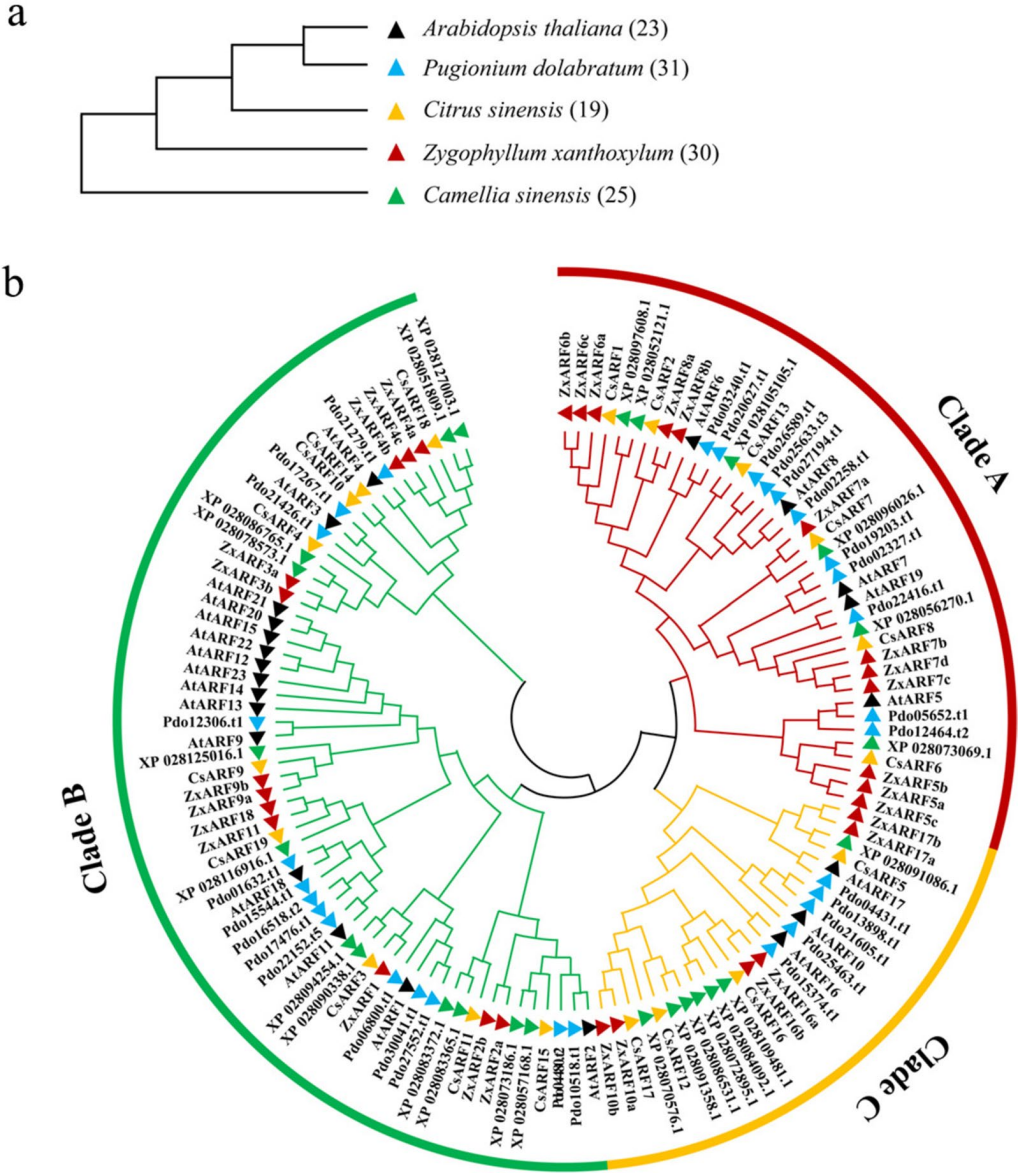
Phylogenetic trees of 3 mesophytes and 2 xerophytes and their ARF family members. **a** Phylogenetic trees of 3 mesophytes and 2 xerophytes. The evolution analysis of *Z. xanthoxylum, P. dolabratum*, *Ci*. *sinensis*, *Ca*. *Sinensis* and Arabidopsis were performed using the ANGIOSPERM PHYLOGENY WEBSITE. Distinctive species are indicated with different colored solid triangles. **b** Phylogenetic analysis of ARF family members of plants in** a**. ARF amino acids sequences were used for constructing an unrooted tree using the maximum likelihood method with 1000 bootstrap replications by MEGA-X. In total, 128 ARFs were analyzed and divided into 3 Clades (A-C), which were exhibited in red, green, and orange, respectively. Distinctive species and the corresponding proteins are indicated with different colored symbols as Fig. 3a

For additionally evidencing that A-ZxARFs expansion is related to the formation of developed root system of *Z. xanthoxylum*, another unrooted phylogenetic tree was constructed based on ARF protein sequences from diploid xerophytes (*Z. xanthoxylum* and *Pugionium dolabratum*) and diploid mesophytes (*Camellia. sinensis*, *Ci. sinensis*, and Arabidopsis) (Fig. 3a, b). All these plants shared an ancient (130–140 Mya) triploidization event [35], and *Z. xanthoxylum*, *P. dolabratum* and *Ca. sinensis* underwent another independent whole-genome duplication (WGD) event in subsequent evolution, respectively [36, 37]. In addition, while *Ca. Sinensis* and *Ci. sinensis* are also shrubs and their shoot sizes are similar to that of *Z. xanthoxylum*, their root systems are less developed than that of *Z. xanthoxylum* [27, 38, 39]. Arabidopsis is the best studied model plant. Thus, these plants are suitable for comparative analysis to explore the relationship between the evolution of A-ARFs and root development.

Here, the ARF family members of *Ca. sinensis* (25) were further complemented base on the study of Xu et al. [40]. And the ARF family members of *P. dolabratum* were firstly identified in this study. ARFs in *Ci. sinensis* were obtained from Li et al. [10]. We found that these ARFs were also clustered into A-C 3 Clades (Fig. 3b). The number of A-ARFs from *Z. xanthoxylum*, *P. granatum*, Arabidopsis, *Ca. sinensis*, and *Ci. Sinensis* were 12, 11, 5, 6, and 6, respectively (Fig. 3b and Table 3), which were consistent with the root characteristics of these plants [27, 36, 38, 39].

**Table 3:** The number of ARF family proteins in mesophytes and xerophytes

Species	Clade A	Clade B	Clade C
Arabidopsis	5	15	3
*P. dolabratum*	11	14	6
*Z. xanthoxylum*	12	12	6
*Ca. sinensis*	6	12	7
*Ci. sinensis*	6	9	4

### Analysis of chromosomal localization and gene replication events of *ZxARFs*

**Fig. 4:**
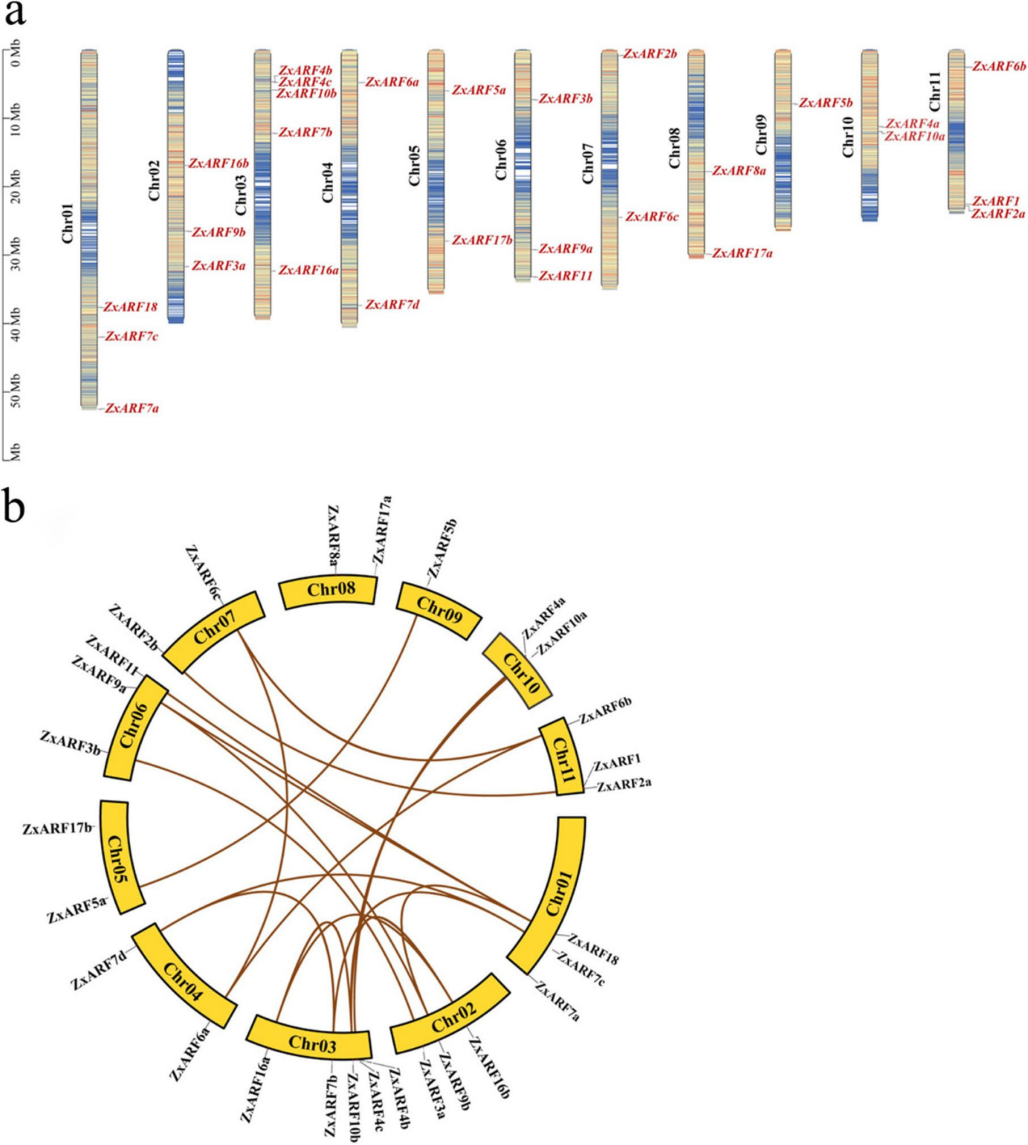
Chromosomal location and syntenic analyses of *ZxARFs*. **a** Chromosomal location of *ZxARFs*. The chromosomal position of each *ZxARF* gene was mapped according to the *Z. xanthoxylum* genome. The chromosome number was indicated at the left side of each chromosome using black characters. The *ZxARFs* gene number was indicated at the right side of each chromosome using red characters. The scale bar represents the length of the chromosomes, and the units is in megabases (Mb). **b** Syntenic analyses of *ZxARFs*. The syntenic relationships of *ARF* genes in *Z.xanthoxylum* were built with MCScanX program by TBtools. The putative orthologous *ZxARF* genes were presented in brown. The chromosome numbers and gene names are indicated in and around the circle, respectively

The distribution of *ZxARFs* on chromosomes were mapped based on the genomic database. Expect for 2 genes lie within unassembled, 28 *ZxARFs* are located on 11 chromosomes of *Z. xanthoxylum* randomly (Fig. 4a). Five *ZxARFs* are located on chromosome 3, three on chromosomes 1, 2, 6 and 11, two on chromosomes 4, 5, 7, 8 and 10, and one on chromosome 9, respectively (Fig. 4a).

To further infer the phylogenetic mechanisms and potential gene duplication events of *ZxARFs*, the synteny relationships and Ks value among the orthologous *ZxARF* genes were performed (Fig. 4b and Table 4). The Ks value of the first and second WGD of *Z. xanthoxylum* range from 0.80–1.16 and 0.22–0.52, respectively. Our results indicated that most of the *ZxARF* genes were amplified by WGD or chromosomal fragment duplication events (Fig. 4). By comparing the Ks values between the orthologous *ZxARFs* gene pairs and WGD of *Z. xanthoxylum*, it was found that the ancestral genes of *ZxARF7c* and *ZxARF7d* were generated by the ancient WGD, and *ZxARF5b*, *ZxARF6b* and *ZxARF7d* were generated by the recent WGD (Table 4).

**Table 4:** Ks values of orthologous *ZxARF* gene pairs

Gene 1	Gene 2	Ks	Gene 1	Gene 2	Ks
*ZxARF5a*	*ZxARF5b*	0.36	*ZxARF9b*	*ZxARF9a*	0.38
*ZxARF6c*	*ZxARF6b*	0.34	*ZxARF10b*	*ZxARF10a*	0.36
*ZxARF7c*	*ZxARF7d*	0.42	*ZxARF16b*	*ZxARF16a*	0.36
*ZxARF2b*	*ZxARF2a*	0.43	*ZxARF7b*	*ZxARF7c*	1.00
*ZxARF3a*	*ZxARF3b*	0.38	*ZxARF7b*	*ZxARF7d*	1.00
*ZxARF4b*	*ZxARF4a*	0.35	*ZxARF18*	*ZxARF11*	1.10

### Expression levels of *A-ZxARFs* in *Z. xanthoxylum* root systems and their expression analysis in response to osmotic stress

**Fig. 5:**
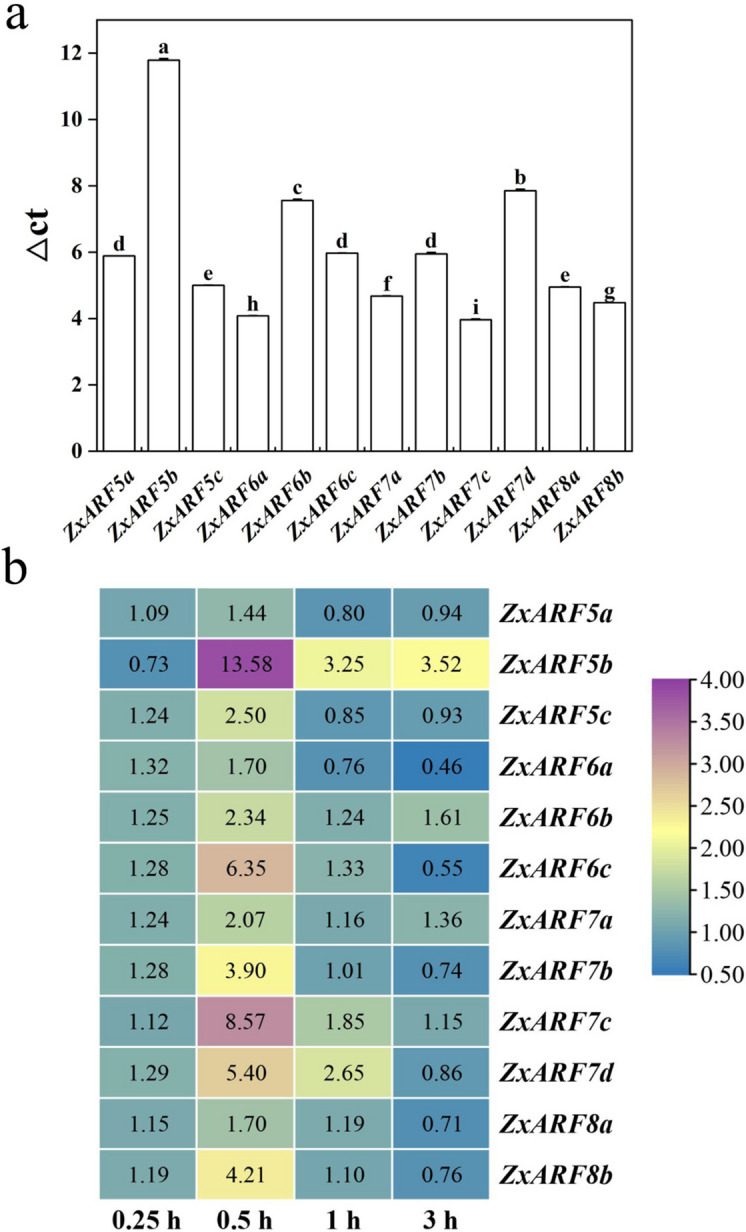
Expression analysis of *A-ZxARFs* in *Z. xanthoxylum* root. **a** Relative transcript abundance profiles of *A-ZxARFs* in roots of *Z. xanthoxylum*. The individual gene names are indicated at the bottom. Different letters on the bars indicate significant difference (*P* < 0.05; n > 3 plants per column). **b** Expression pattern analysis of *A-ZxARFs* in roots of *Z. xanthoxylum* under osmotic stress (Simulated drought) treatment. Different colors in map represent different transcript abundance values. The scale bar on the right indicates relative expression levels. Time of osmotic stress treatments are indicated at the bottom. The individual gene names are indicated on the right

Expression pattern of a gene is correlated with its function. To verify whether these *A-ZxARFs* were involved in the growth and development of root, their expressions in *Z. xanthoxylum* root system were confirmed by RT-qPCR. As shown in Fig. 5a, the Δct value of almost all *A-ZxARFs* were less than 10, indicating that they have high expression levels in the *Z. xanthoxylum* root system. Among them, the highest expressed gene was *ZxARF5b* and the lowest expressed gene was *ZxARF7c* (Fig. 5a).

Extensive research has shown that various environmental signals are integrated into changes in auxin homeostasis, redistribution, and signaling [41]. To further confirm the function of *A-ZxARF* genes, we analyzed their expression levels under osmotic stress treatment (simulate drought stress) (Fig. 5b). Almost all *A-ZxARFs* were induced by osmotic stress treatment and peaked at 30 min (Fig. 5b). Among them, the expression level of *ZxARF5b*, *ZxARF7c*, *ZxARF6c* and *ZxARF7d* showed the most significant changes, with 13.58, 8.57, 6.35 and 5.40-fold up-regulation at 30 min, respectively (Fig. 5b). Therefore, we hypothesized that drought stress signals may induce the expression of *A-ZxARFs* to regulate root development of *Z. xanthoxylum*, resulting in the formation of the developed root system.

### Protein domain analysis of ZxARFs

**Fig. 6:**
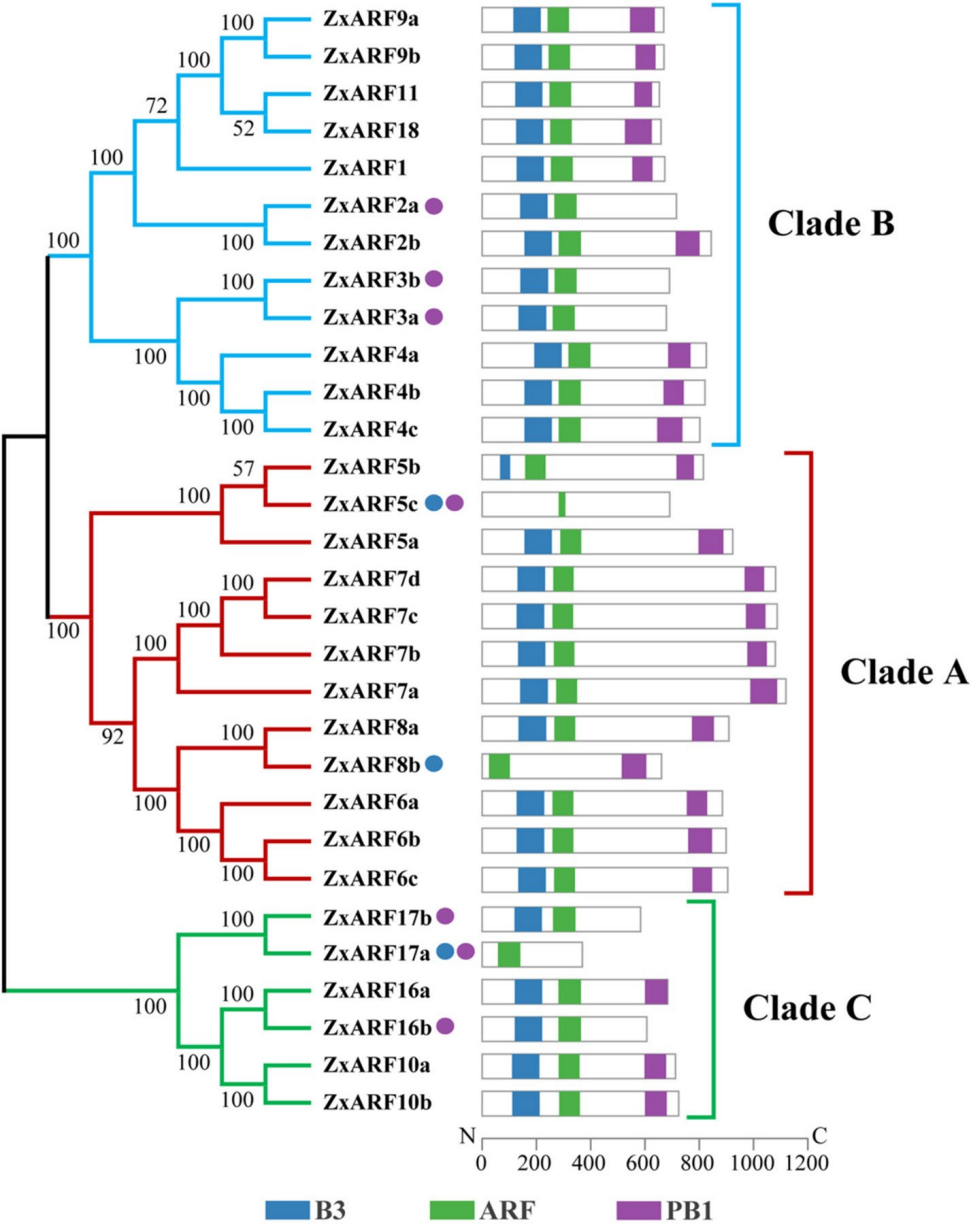
Analyses of phylogenetic relationships and conserved domains of ZxARFs*.* 30 ARFs of *Z. xanthoxylum* were used for constructing an unrooted tree using the maximum likelihood method with 1000 bootstrap replications by MEGA-X. These ZxARFs divided into 3 Clades (A-C), which were exhibited in red, blue, and green, respectively. Lengths of each domain are displayed proportionally. The blue boxes represent B3 domains, green boxes represent ARF domains, and purple boxes represent PB1 domains. The blue and purple solid circle represents the absence of B3 domain and PB1 domain, respectively

Protein structure is vital to determine the function of a protein and its interactive network. Thus, domains of ZxARF proteins were identified and analyzed using NCBI Conserved Domain Database. The results showed that most of the ZxARFs shared similar domain compositions in the same groups (Fig. 6), suggesting functional similarities within the same subfamily.

All the identified ZxARFs harbored ARF domains, but 11 members (ZxARF2a, ZxARF3a, ZxARF3b, ZxARF4c, ZxARF5c, ZxARF10a, ZxARF10b, ZxARF16a, ZxARF16b, ZxARF17a and ZxARF17b) lacked the PB1 domain, 3 members (ZxARF5c, ZxARF8b and ZxARF17a) lacked the typical B3 DNA-binding domains (Fig. 6). As one of the oldest members of ZxARF proteins, there are no DBD or CTD in ZxARF17a. In addition, ZxARF5b and ZxARF8b (A-ZxARFs) have lost the B3 DNA-binding domain partly and completely, respectively, but their PB1 domain was retained (Fig. 6), indicating that these proteins may have specific functions*.*

### *ZxARF7c* and *ZxARF7d* promote LR growth and development

**Fig. 7:**
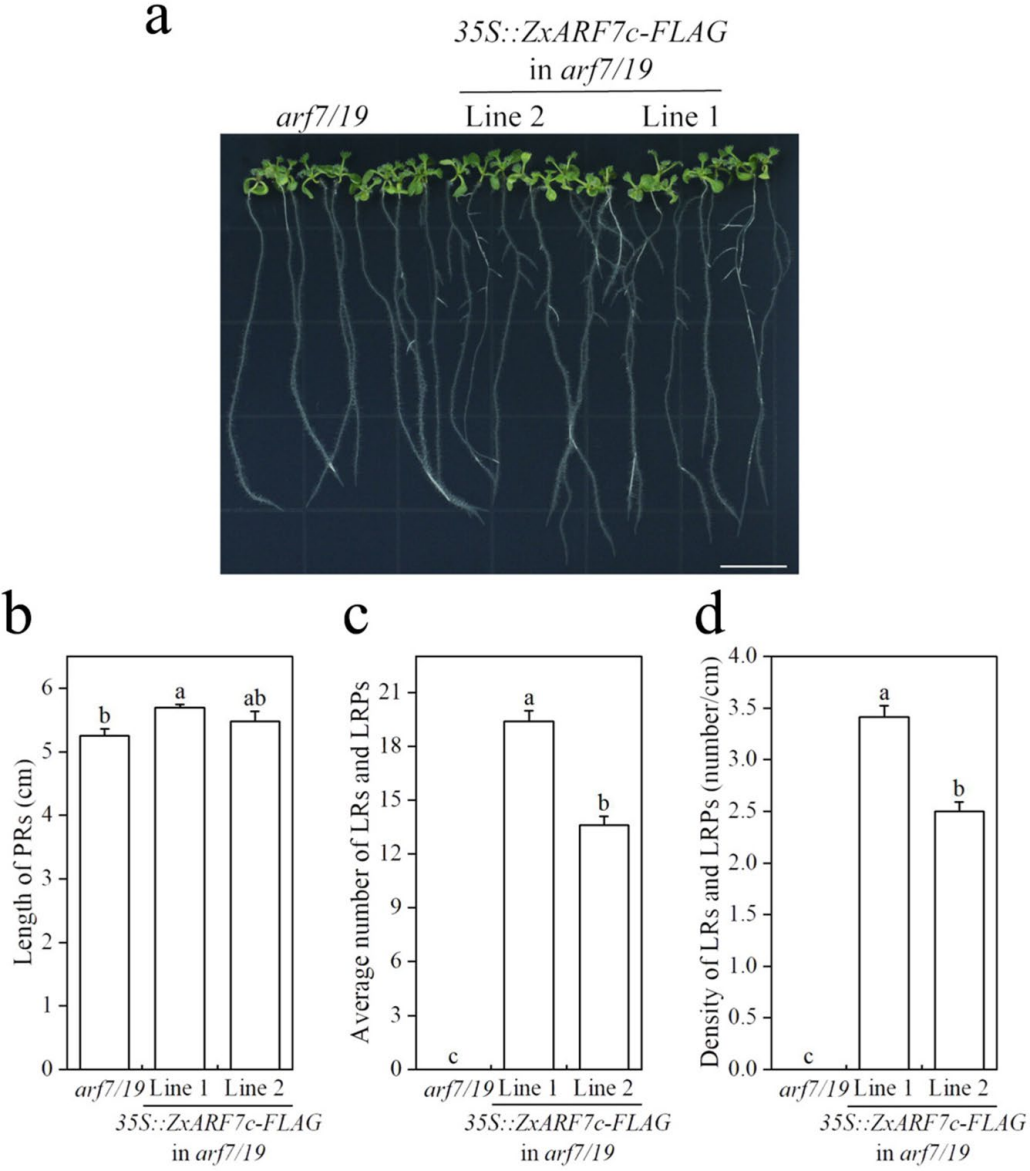
Root phenotype analysis of *35S::ZxARF7c-FLAG* in *arf7/19*-related seedlings. **a** Phenotypes of *arf7/19* and *ZxARF7c-FLAG* overexpression lines grow in light for 10 d after germination. Scale bar = 1 cm; **b-d** Statistical analysis of PRs length (**b**) and LRs and LRPs number (**c**) and density of LRs and LRPs (**d**) of plants in **a**. Different letters on the bars indicate significant difference (*P* < 0.05; n > 10 plants per column)

**Fig. 8:**
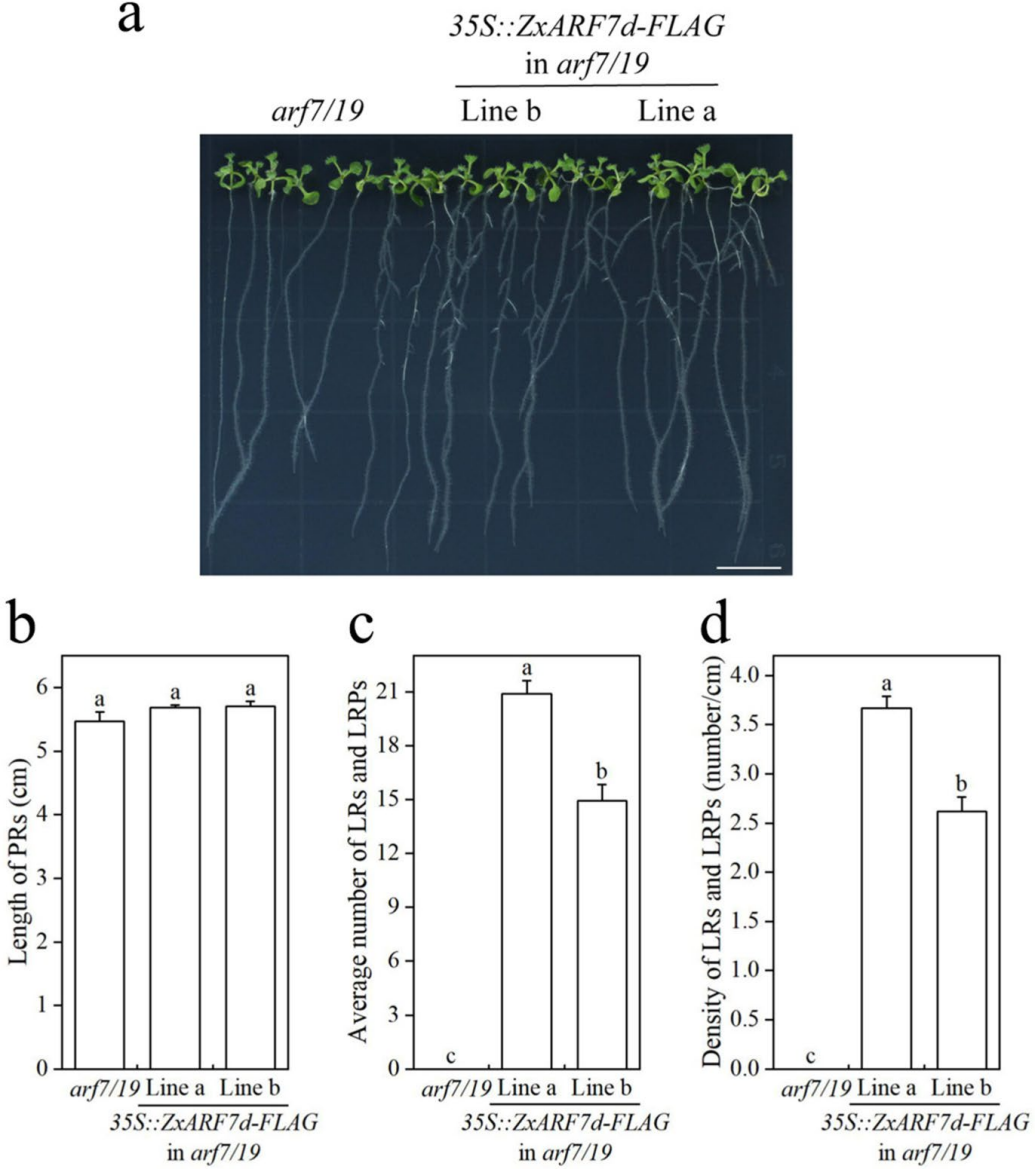
Root phenotype analysis of *35S::ZxARF7d-FLAG* in *arf7/19*-related seedlings. **a** Phenotypes of *arf7/19* and *ZxARF7d-FLAG* overexpression lines grow in light for 10 d after germination. Scale bar = 1 cm; **b-d** Statistical analysis of PRs length (**b**) and LRs and LRPs number (**c**) and density of LRs and LRPs (**d**) of plants in **a**. Different letters on the bars indicate significant difference (*P* < 0.05; n > 10 plants per column)

**Fig. 9:**
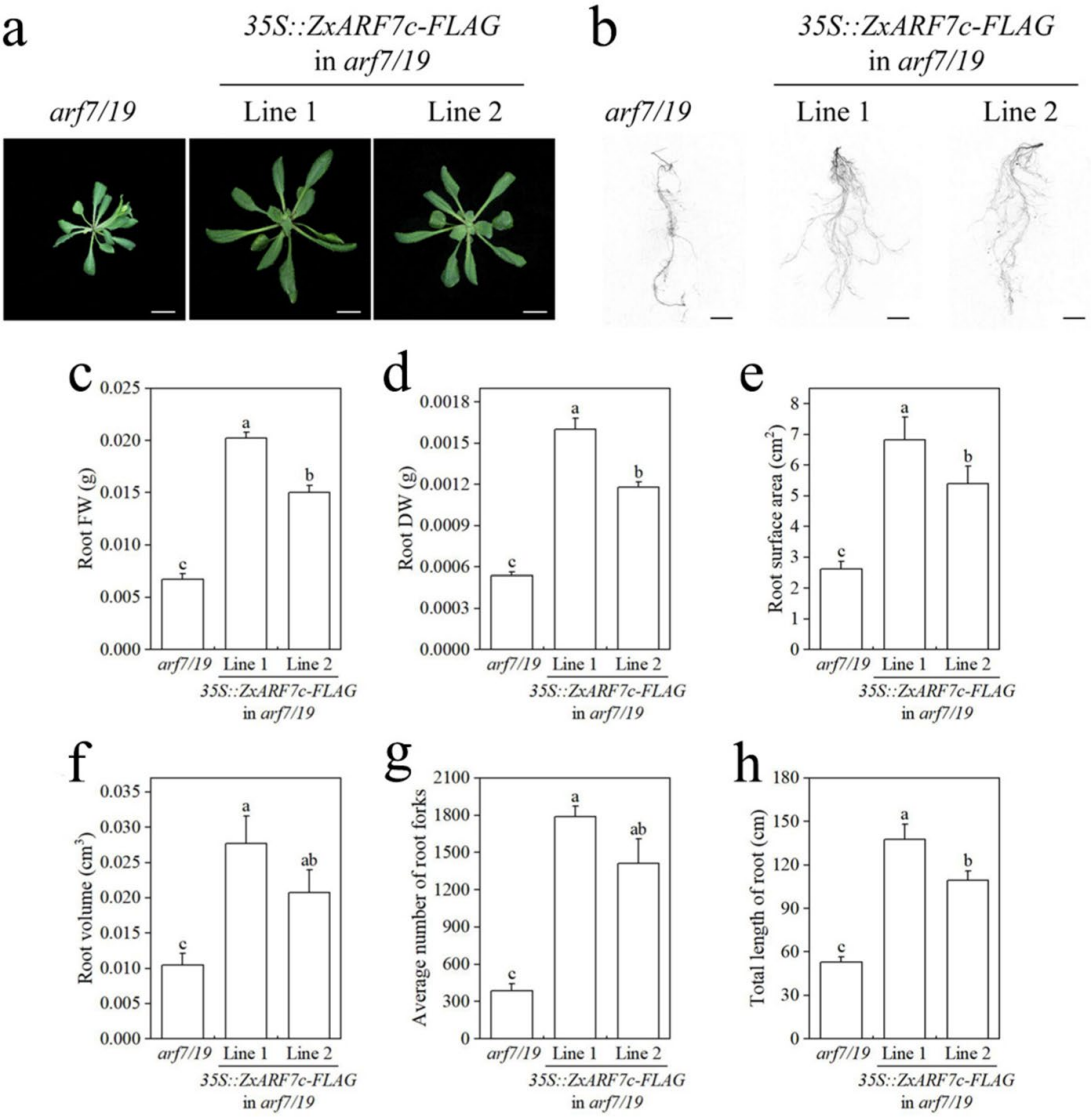
Phenotypes analysis of *35S::ZxARF7c-FLAG* in *arf7/19*-related seedlings. **a** Phenotypes of rosette. Scale bar = 1 cm; **b** Phenotypes of root system. Scale bar = 1 cm; **c-f** Statistical analysis of root fresh weight (**c**) and root dry weight (**d**) and root surface area (**e**) and root volume (**f**) and root forks (**g**) and total length of root system (**h**) of plants in **b**. Different letters on the bars indicate significant difference (*P* < 0.05; n > 10 plants per column)

**Fig. 10:**
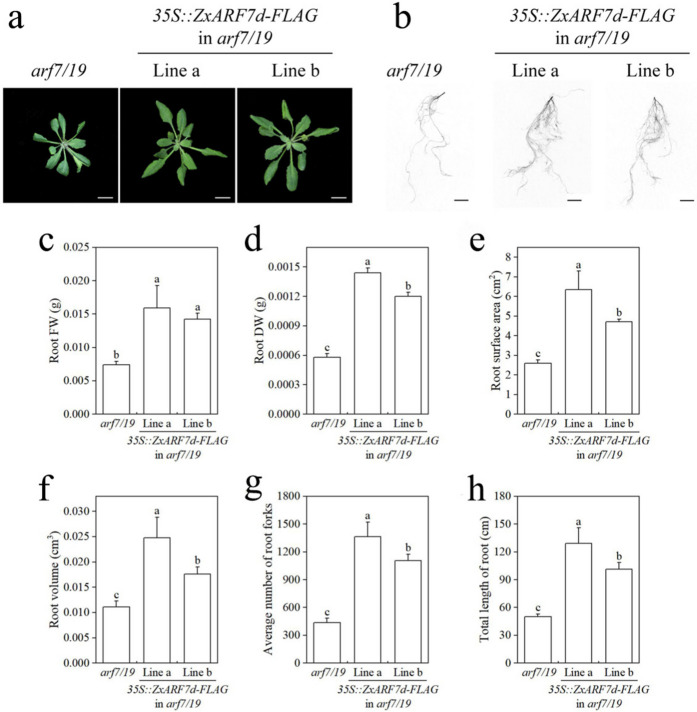
Phenotypes analysis of *35S::ZxARF7d-FLAG* in *arf7/19*-related seedlings. **a** Phenotypes of rosette. Scale bar = 1 cm; **b** Phenotypes of root system. Scale bar = 1 cm; **c-f** Statistical analysis of root fresh weight (**c**) and root dry weight (**d**) and root surface area (**e**) and root volume (**f**) and root forks (**g**) and total length of root system (**h**) of plants in **b**. Different letters on the bars indicate significant difference (*P* < 0.05; n > 10 plants per column)

*AtARF7/19* plays a key role in LR formation in Arabidopsis, and *ZxARF7c* and *ZxARF7d* are highly homologous to *AtARF7/19* (Fig. 1). To characterize the function of *ZxARF7c* and *ZxARF7d* in LR growth and development, the coding sequence (CDS) of *ZxARF7c* and *ZxARF7d* were cloned. Stabilized *ZxARF7c*-OE lines (Line a and Line b) and *ZxARF7d*-OE lines (Line 1 and Line 2) were generated by overexpressing *ZxARF7c* and *ZxARF7d* under a 35S promoter in the *arf7 arf19* double mutant of Arabidopsis, respectively. RT-PCR assay confirmed that *ZxARF7c* and *ZxARF7d* transcripts were obviously increased in *ZxARF7c*-OE and *ZxARF7d*-OE lines and no detectable in *arf7 arf19* double mutant seedling (Fig. S1-S3). The results showed that defect of LR development of *arf7 arf19* seedlings are rescued completely by *ZxARF7c* and *ZxARF7d* overexpression, respectively (Figs. 7 and 8). Similarly, the rosette of *arf7 arf19* adult plants were rescued via *ZxARF7c* and *ZxARF7d* overexpression, respectively (Figs. 9 and 10).

In addition, the transgenic plants had larger root surface area and root volume, more root forks and longer total root length than the *arf7 arf19* background plant (Figs. 9d-h and 10d-h). Also, fresh weight and dry weight of the root from these transgenic plants are higher compared to that of *arf7 arf19* mutant (Figs. 9b-c and 10b-c). These collective data indicates that *ZxARF7c* and *ZxARF7d* play a positive role in LR formation and growth.

## Discussion

It has been reported that *ARF* gene family play vital roles in root development and response to abiotic stresses [23–25]. However, the functions of xerophytes *ARF* genes are remained poorly characterized. In the present study, we first identified 30 ZxARF proteins from the recent genome sequencing of the desert xerophytes *Z. xanthoxylum* (Table 1). Furthermore, a comprehensive information of *ZxARF* gene family has been provided and analyzed.

ARF family are divided into A, B, and C Clades in land plants [13]. Finet et al. [13] inferred that Clade A and C can probably be traced back to the origin of land plant, since they include bryophyte sequences. However, Kato et al. [7] have identified a B-ARF from *M. polymorpha*. Meantime, Martin-Arevalillo et al. [14] found that the most ‘ancient’ ARF homologue is *C. atmophyticus* ARF (CaARF), which belongs to Clade C. Similarly, in this study, we identified that the only one ARF protein from *C. braunii* is also a C-ARF (Fig. 2b). Therefore, we may conclude that the first ARF proteins originated from algae, which probably be a C-ARF.

As a super-xerophyte, *Z. xanthoxylum* has formed highly developed root system network dominated by lateral roots [27]. The root systems of mesophytes, such as *Ca. sinensis* and *Ci. sinensis*, are less developed [27, 38, 39]. By constructing phylogenetic tree of ARFs from *Z. xanthoxylum* and these mesophytes including *Ca. sinensis* and *Ci. sinensis* at the same evolutionary stage (Fig. 3b), it is noteworthy that the number of A-ZxARFs is far greater than that of A-ARFs from these mesophytes. In addition, as another super-xerophyte, *P. dolabratum* also harbours as many A-ARFs numbers as *Z. xanthoxylum* (Fig. 3b). Given that A-AtARFs play vital roles in every stage of lateral roots development in Arabidopsis [15], we concluded that A-ARFs from other plants also play important roles in the lateral roots development, and the massive expansion of A-ZxARFs may be the important mechanism for the formation of the developed root system of the xerophyte *Z. xanthoxylum*.

In addition, based on the fossil evidence and the root anatomy of extant vascular plants, three root-evolution events were proposed during vascular plant evolution, giving rise to the extant bifurcating roots in lycophytes, adventitious/lateral roots in euphyllophytes (ferns and seed plants), and primary roots in seed plants [42]. According to the phylogenetic tree of ARFs from alga to higher plant species, we found that ARF10/16 in Clade C is restricted to phanerogams. Given that ARF10/16 are involved in the root development in Arabidopsis [22], we speculate that these proteins of ARF10/16 branch may play important roles in root evolution in seed plants rather than lycophytes.

A typical ARF protein contains three domains, designated as DBD, MR and PB1 [4]. Most ZxARFs were characterized by the classic domains, while 11 members lacked the PB1 domain and 3 members lacked the typical B3 DNA-binding domains (Fig. 6). Especially, ZxARF5b and ZxARF8b (A-ZxARFs) have lost the B3 domain partly and completely (Fig. 6).Ulmasov et al. [5] found that ARFs with Q-Rich MRs can activate transcription on TGTCTC AuxREs in the absence of a DBD. Similarly, Tiwari et al. [43] found that the ARF MR can function as an AD or an RD when fused to a heterologous DBD, and targeted to non-auxin response gene promoters. Therefore, ZxARF5b and ZxARF8b may function as activators and compete with other A-ZxARFs to bind the transcriptional repressor ZxAux/IAAs, which help release these A-ZxARFs from ZxAux/IAAs-ZxARFs dimers to trigger transcription of downstream genes, and then initiate lateral root formation. Due to ZxARF5c only has a truncated ARF domain, it might be a pseudogene, or express and function at specific developmental stages under special conditions. The exact contributions of these ARFs remain to be elucidated, especially for those that lack one or even two domains.

## Conclusions

In this study, we found that A-ZxARFs had expanded largely in the process of evolution, which may be the important mechanism for the formation of the developed root system of *Z. xanthoxylum*. This assumption was further confirmed by over-expression assay in Arabidopsis. Our studies will pave the way for further understanding the potential role of *A-ZxARF* genes in plant root development.

## Materials and methods

### Plant materials and growth conditions

Seeds of *Z. xanthoxylum* were collected from the Minqin (38°03′N, 101°49′E; elevation 1371 m) region, which were identified by Minqin desert botanical garden (http://www.nfgrp.cn/data/list/resource_detaillist.html), and stored in the Key Laboratory of Grassland Livestock Industry Innovation, Ministry of Agriculture and Rural Afairs, Lanzhou, China. The corresponding voucher specimen (Chase 1700 (K)) has been deposited in stored in School of Life Science, Shihezi University [44]. *Z. xanthoxylum* plants were grown under long-day conditions (16 h/8 h light/dark; 600 μmol m^−2^ s^−1^ light intensity; 40%-65% relative humidity; 28 ± 2 ℃) for 3 weeks. For the drought treatment, irrigation was 1/2 Hoagland solution containing 300 mM Mannitol. Control (CK) plants were all irrigated with 1/2 Hoagland solution. Roots were sampled after 15 min, 30 min 1 h and 3 h treatment. All samples were immediately frozen in liquid nitrogen after harvesting and stored at -80 °C until use.

The T-DNA insertion alleles *arf7 arf19* (CS24630) was obtained from the Arabidopsis Biological Resource Center (ABRC), and was identified and propagated by our group before [16]. For plate assay, Arabidopsis seeds were placed on 1/2 Murashige and Skoog (MS) medium containing 1% (w/v) sucrose and cold-treated at 4℃ for 4 d, and then transferred to long-day conditions (16 h/8 h light/dark; 100 μmol m^−2^ s^−1^ light intensity; 40%-65% relative humidity; 22℃ ± 2℃) to grow for 10 d [16]. For soil culture assay, Arabidopsis seeds were cold-treated at 4℃ for 4 d, and then sown in sterilized soils and grown in long-day conditions as above.

### Identification of plant ARF family members

The annotated protein sequences of *C. braunii*, *S. moellendorfii*, *A. trichopoda* and *Ca. sinensis* were all obtained from National Center for Biotechnology Information (NCBI, https://www.ncbi.nlm.nih.gov/) database resources. The annotated protein sequences of *P. dolabratum* were obtained from Hu et al. [36]. The annotated protein sequences of *Z. xanthoxylum* were obtained from our laboratory and available in the National Center for Biotechnology Information BioProject database (https://www.ncbi.nlm.nih.gov/bioproject/?term=PRJNA933961). The ARF amino acids sequences of Arabidopsis were obtained from The Arabidopsis Information Resource (TAIR, https://www.arabidopsis.org/). The ARF amino acids sequences of *C. atmophyticus*, *M. polymorpha*, *P. patens*, *G. biloba* and *Ci. sinensis* were obtained from previous studies [7, 10, 14, 33, 34].

Twenty-three ARF amino acids sequences of Arabidopsis were used as queries against protein database of *C. braunii*, *S. moellendorfii*, *A. trichopoda*, *Ca. Sinensis*, *P. dolabratum* and *Z. xanthoxylum*, respectively. The possible ARF proteins were further characterized by BLAST analysis via TAIR database. Finally, all identified ARFs were further validated by a conserved domain search using the CDD (http://www.ncbi.nlm.nih.gov/cdd/) and Pfam (http://pfam.sanger.ac.uk/) databases. Candidate proteins without ARF domain were removed. All ARF amino acids sequences used in this study were listed in Table S1.

### Establishment of phylogenetic trees for plants and ARF proteins

Based on ANGIOSPERM PHYLOGENY WEBSITE (https://www.mobot.org/MOBOT/research/APweb/), we established plant phylogenetic trees of the representative species in plant evolutionary history including *C. atmophyticus*, *C. braunii*, *M. polymorpha*, *S. moellendorfii*, *P. patens*, *G. biloba*, *A. trichopoda*, Arabidopsis and *Z. xanthoxylum*. Also, we established plant phylogenetic trees of the mesophytic and xerophytic species including Arabidopsis, *Ca. Sinensis*, *Ci. Sinensis*, *P. dolabratum* and *Z. xanthoxylum*. Phylogenetic analysis of ARF proteins were carried out according to the maximum likelihood method using the Clustal W and MEGA X software packages, and support for each node was assessed by performing a bootstrap analysis with 1000 replicates [45].

### Protein properties and sequences analyses

The amino acid properties, molecular weights, and isoelectric points (pI) of ZxARFs were analyzed using ProtParam online software (http://web.expasy.org/protparam/). The subcellular localizations were predicted by Plant-mPLoc online software (http://www.csbio.sjtu.edu.cn/bioinf/plant-multi/). The structural features of the *ZxARF* genes were identified using Gene Structure View (Advanced) program in TBtools (Version 1.0986853) [46] based on the genome and coding sequences (CDS) of *ZxARFs*. The conserved domains of ZxARF proteins were analyzed using the CDD and Pfam databases, and the results were further visualized and modified by Gene Structure View (Advanced) program in TBtools (Version 1.0986853).

### Chromosomal location and gene duplication of *ZxARF* family genes

The chromosomal locations of *ZxARFs* were determined based on the positional information obtained from the *Z. xanthoxylum* genome database, and the results were further visualized by Gene Location Visualize (Advanced) program in TBtools (Version 1.0986853). The syntenic relationships of *Z. xanthoxylum* genome was generated with One Step MCScanX program in TBtools (Version 1.0986853), and the syntenic relationships among the orthologous *ZxARF* genes were highlighted. The Ks (synonymous substitution rate) was computed between pairs of genes identified as homeologous by Simple Ka/Ks Calculator program in TBtools (Version 1.0986853).

### Quantitative real-time PCR analysis

An RNAprep Pure Plant Kit (Tiangen) was used to isolate total RNA of leaves from each plant and first-strand cDNA was synthesized from the RNA by using a PrimeScript™ RT Master Mix Kit (TaKaRa). qRT-PCR was performed in triplicate on three bioreplicates using Power SYBR™ Green Master Mix (TaKaRa Biotechnology, China) on a StepOne Real-Time PCR Thermocycler (Applied Biosystems). All kits were used according to the manufacturer instructions. Primer-BLAST (https://www.ncbi.nlm.nih.gov/tools/primer-blast) was used for primer design according to the following criteria: PCR amplicon lengths of 80–120 bp, Tm of 60 ± 1℃ and GC contents of 45–60%. The primers employed were listed in Table S2. The comparative cycle threshold method (ΔΔCT) was used to calculate relative expression levels, and *ZxACTIN* (GenBank: *EU019550.1*) from *Z. xanthoxylum* was used as the reference gene.

### Vector construction and Arabidopsis plant transformation

Transformation constructs were obtained with Gateway technology (Invitrogen, Carlsbad CA, USA). The CDS of *ZxARF7c* and *ZxARF7d* were amplified by polymerase chain reaction (PCR) and introduced into a *pDONR™/ZEO* vector using Gateway® BP Clonase™ II Enzyme Mix to generate entry clones. The resulting entry clones were used to transfer target sequences into destination vectors with a *Cauliflower mosaic virus 35S* promoter and *FLAG* tag (*pBIB*-*35S*-*GWR*-*FLAG*) by LR recombination reaction using Gateway LR clonase II enzyme mix (Invitrogen). These constructs were introduced into *Agrobacterium tumefaciens* strain *GV3101* and used for *Agrobacterium*-mediated gene transfer under *arf7 arf19* backgrounds [47]. Transgenic plants were selected by 0.01% BASTA [16]. The expressions of *35S::ZxARF7c-FLAG* and *35S::ZxARF7d-FLAG* were confirmed with semi-quantitative reverse-transcription-polymerase chain reaction (RT-PCR). And two independent transgenic lines of each gene were used for further analyses, respectively. Primers for gene clones were listed in Table S2.

### Semi-quantitative reverse-transcription-polymerase chain reaction

An RNAprep Pure Plant Kit (Tiangen) was used to isolate total RNA of leaves from each plant and first-strand cDNA was synthesized from the RNA by using a PrimeScript™ RT Master Mix Kit (TaKaRa) according to the manufacturer’s instructions. *AtACTIN* (TAIR: *At3g18780*) was used as the reference gene. Semi-quantitative PCR was performed in 18 cycles for *AtACTIN2* and 35 cycles for *ZxARF7c* and *ZxARF7d*. The PCR products were analyzed on 2% agarose gels. Equal loading of each amplified gene sequence was determined with the *AtACTIN2* PCR product. All amplifications were performed at least three times. Primers for RT-PCR assay were listed in Table S2.

### Root system traits measurements

Arabidopsis seedlings grown on 1/2 MS solid medium for 10 d. Length of the primary roots was measured by Image J (Version 1.42q, NIH) software. Number of LRP/LR was counted with an Olympus light microscope. The density of LRP/LR was indicated via dividing LRP/LR number by the primary root length. The number of each replicate for per genotype was carried out at least 10 plants. For 3-week-old Arabidopsis plants grown in pots, the roots were gently washed to separate and then placed on a transparent tray filled with water. The root trays were scanned with an Epson Perfection V700 scanner to obtain a grayscale TIFF image. Subsequently, the images were analyzed by using the WinRHIZO Pro image processing system (Regent Instruments Inc., 2672 Chemin Sainte-Foy, Quebec City, Quebec G1V 1 V4, Canada) to obtain root surface area, root volume, root forks, and total root length. Next, roots were dried with absorbent paper to get their fresh weight, and to obtain their dry weight after being placed at 75℃ for 48 h. The number of replicates per genotype was carried out at least 6 plants. All photographs were taken with a digital camera.

### Supplementary Information

**Additional file 1. **

**Additional file 2. **

**Additional file 3. **

## Data Availability

The datasets analyzed during the current study are included within the article and its supplementary information files. The annotated protein sequences of *P. dolabratum*, *C. braunii*, *S. moellendorfii*, *A. trichopoda* and *C. sinensis* were all obtained from National Center for Biotechnology Information (NCBI, https://www.ncbi.nlm.nih.gov/) database resources. The whole genome sequences of *Z. xanthoxylum* are available in the NCBI BioProject database (https://www.ncbi.nlm.nih.gov/bioproject.) under the accession numbers PRJNA933961.

## Abbreviations

ARF: Auxin response factor
TFs: Transcription factors
TIR1/AFB1-5: Transport inhibitor response 1/auxin signaling F-box protein
DBD: DNA-binding domain
MR: Middle region
CTD: Conserved C-terminal domain
PB1: Phox and Bem1p
HAK5: High affinity K^+^ transporter 5
SUMO: Small ubiquitin-like modifier
WGD: Whole-genome duplication
CDS: Coding sequences
