# Supplementary material for: Evolution and function analysis of auxin response factors reveal the molecular basis of the developed root system of Zygophyllum xanthoxylum
Source: BMC Plant Biol. 2024 Feb 2;24:81. doi: 10.1186/s12870-023-04717-7 (PMC10835889; doi:10.1186/s12870-023-04717-7)
Supplement: Supplementary file 3 — Additional file 3. [file 12870_2023_4717_MOESM3_ESM.docx]

**Supplementary Figures**

**
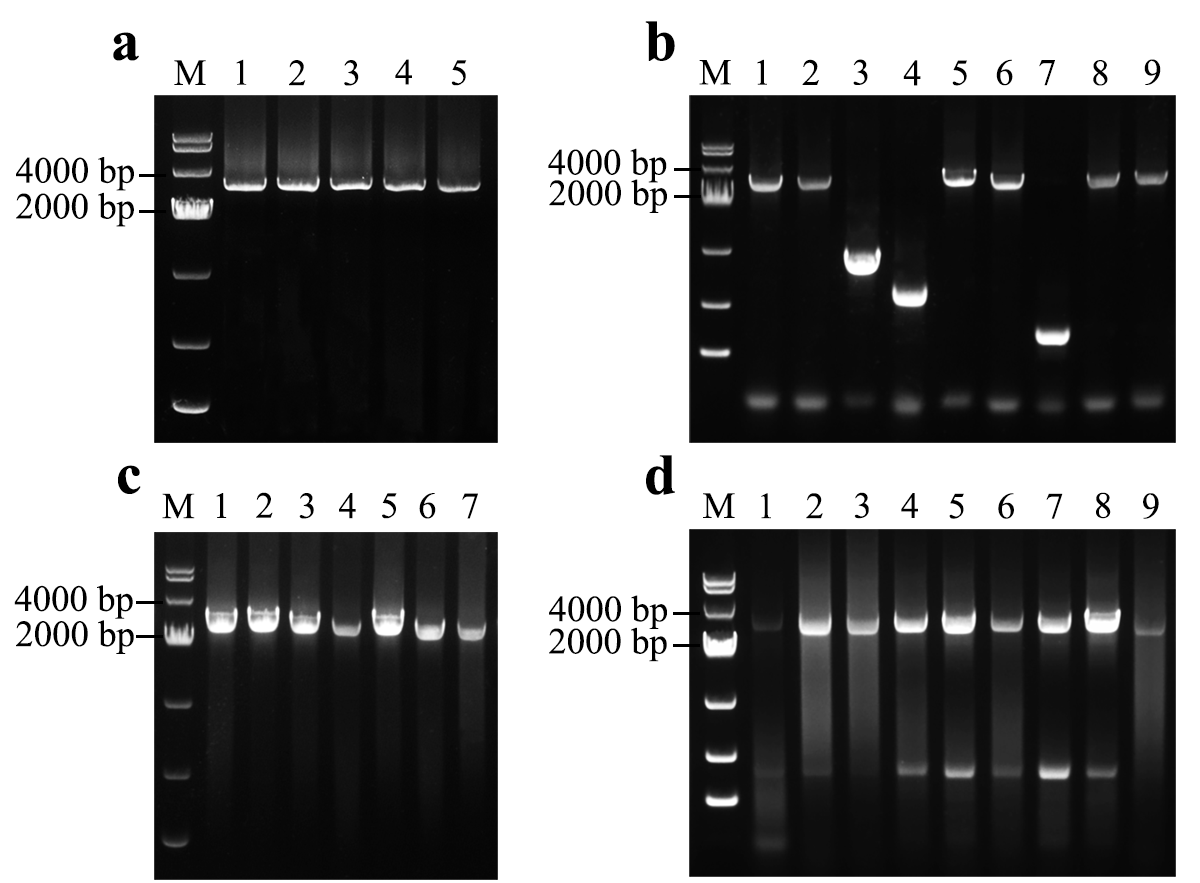
**

**Additional file 1: Fig. S1.** Agarose gel electrophoresis of the PCR product for *ZxARF7c* expression vector construction. A, Cloning of *ZxARF7c*. lanes 1-5: positive clone. B, Bacteria colony PCR identification of BP recombination reactions. lanes 1,2,5,6,8,9: positive strain. C, Bacteria colony PCR identification of LR recombination reactions. lanes 1-7: positive strain. D, Bacteria colony PCR identification of LR plasmid transformed into *GV3101*. lanes 1-9: positive strain. M: 10000 bp Marker.


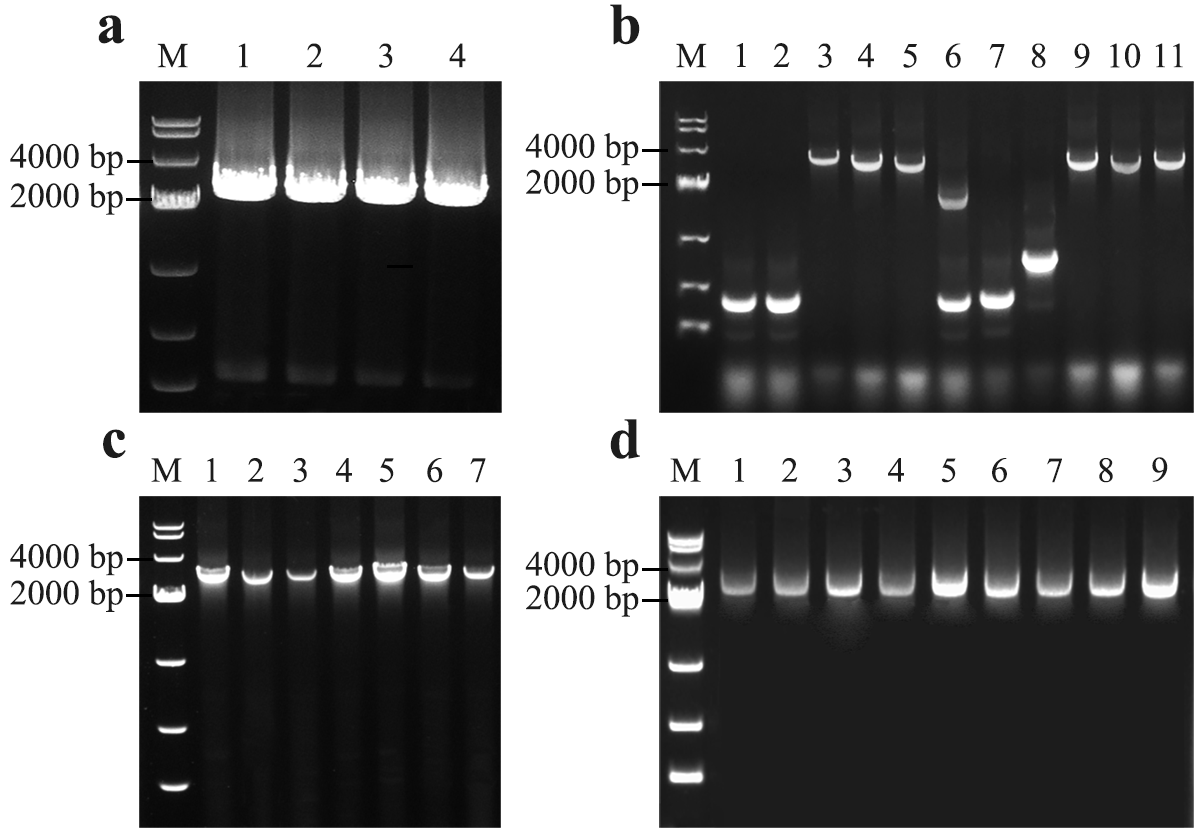


**Additional file 2: Fig. S2.** Agarose gel electrophoresis of the PCR product for *ZxARF7d* expression vector construction. A, Cloning of *ZxARF7d*. lanes 1-4: positive clone. B, Bacteria colony PCR identification of BP recombination reactions. lanes 1,2,3-5,9-11: positive strain. C, Bacteria colony PCR identification of LR recombination reactions. lanes 1-7: positive strain. D, Bacteria colony PCR identification of LR plasmid transformed into *GV3101*. lanes 1-9: positive strain. M: 10000 bp Marker.


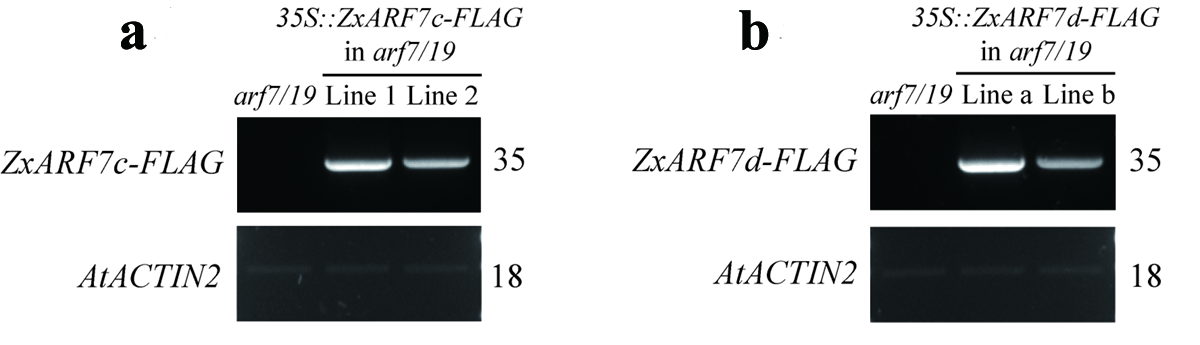


**Additional file 3: Fig. S3.** Relative transcription level analysis of *ZxARF7c-FLAG* and *ZxARF7d-FLAG* in the transgenic Arabidopsis. A, The expression level of *ZxARF7c* in *35S::ZxARF7c-FLAG* in *arf7/19*-related plants; B, The expression level of *ZxARF7d* in *35S::ZxARF7d-FLAG* in *arf7/19*-related plants. *AtACTIN2* was used as the internal control gene.

**
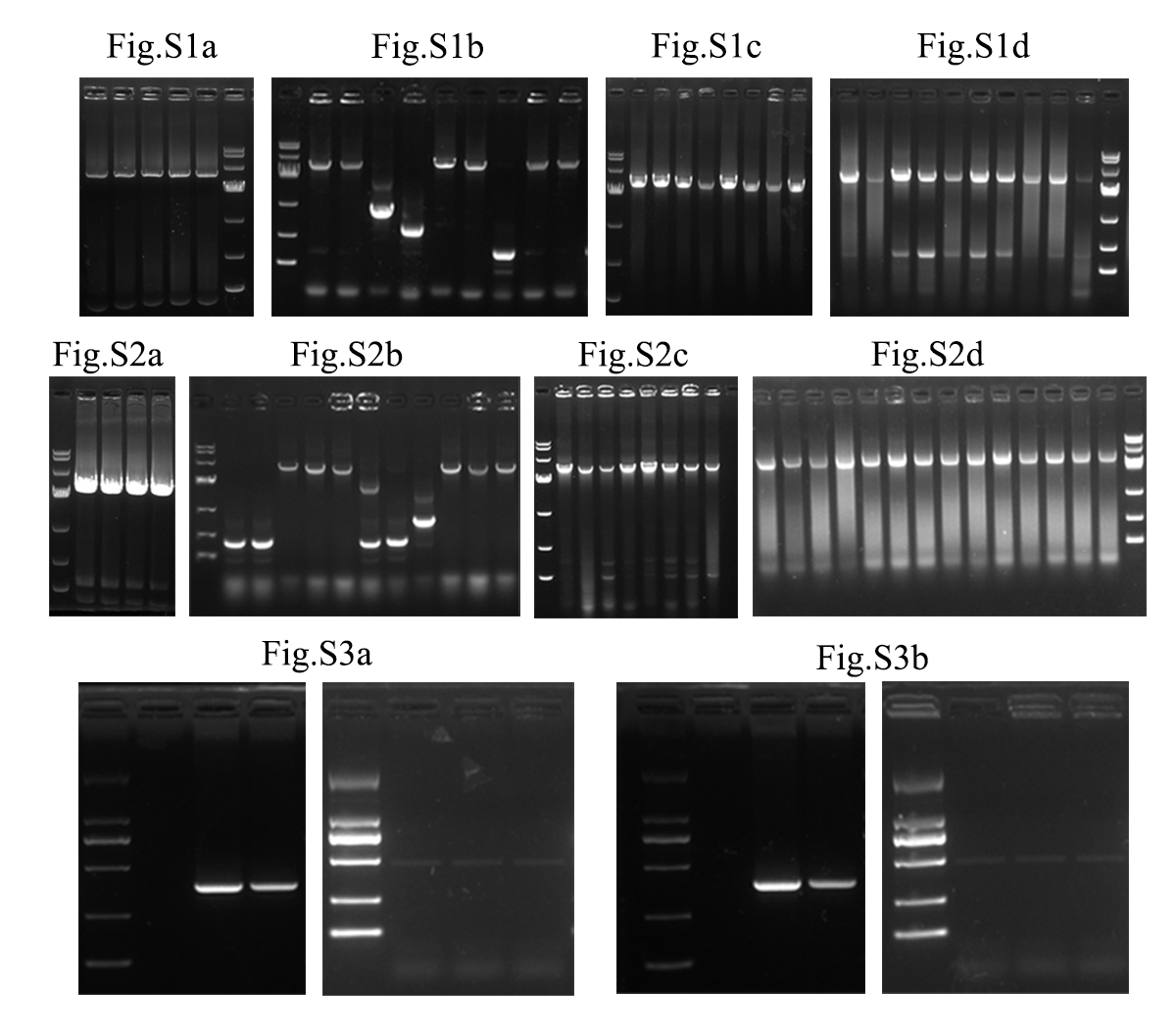
**

**Additional file 6: Fig. S4.** Full uncropped Gels images in this study.
